# Draft Genome Sequence of *Pseudomonas mediterranea* Strain CFBP 5447^T^, a Producer of Filmable Medium-Chain-Length Polyhydroxyalkanoates

**DOI:** 10.1128/genomeA.01260-14

**Published:** 2014-12-24

**Authors:** G. Licciardello, P. Bella, G. Devescovi, C. P. Strano, P. F. Sarris, A. F. Catara, V. Venturi, V. Catara

**Affiliations:** a Science and Technology Park of Sicily, Catania, Italy; b Department of Agriculture and Food Science, University of Catania, Catania, Italy; c Bacteriology Group, International Centre for Genetic Engineering and Biotechnology (ICGEB), Area Science Park, Padriciano, Trieste, Italy; d The Sainsbury Laboratory, John Innes Centre, Norwich Research Park, Norwich, United Kingdom

## Abstract

*Pseudomonas mediterranea* strain CFBP 5447^T^ is a phytopathogenic bacterium isolated from tomato plants affected by pith necrosis disease. Moreover, its ability to produce medium-chain-length polyhydroxyalkanoates (mcl-PHAs) in culture from different carbon sources and valuable microbial products, such as cyclic lipopeptides, has been well documented. Here, we report the first draft genome sequence of this species.

## GENOME ANNOUNCEMENT

*Pseudomonas mediterranea* is phylogenetically close to *Pseudomonas corrugata* and *Pseudomonas brassicacearum* subsp. *brassicacearum* (1, 3). The *P. mediterranea* species type strain CFBP 5447^T^ (our lab ID 9.1) was isolated in Italy from tomato plants affected by pith necrosis (2, 3). One of the characteristics of this species is the conversion of carbon sources, such as biodiesel-derived glycerol, to medium-chain-length polyhydroxyalkanoates (mcl-PHAs) (4, 5), having peculiar filmable properties of potential interest as a softener of other biopolymers (6, 7). It also produces cormycin A and corpeptins, which are cyclic lipopeptides (CLPs) with antimicrobial and biosurfactant activities (8).

Here, we report the draft genome of *P. mediterranea* CFBP 5447^T^, which is the first strain sequenced of this species. The sequencing was performed by BaseClear BV, Leiden, the Netherlands, by using Illumina GAIIx technology combining mate-pair and paired-end libraries. We obtained 9,460,146 pairs of reads representing approximately 176-fold coverage. *De novo* assembly using CLC Genomics Workbench version 5.1 (CLC bio, Denmark) generated 47 contigs (minimum contig size, 336 nucleotides), with a maximum contig size of 991,459 bp. The total length of the contig assembly was 6,311,439 bp, and the *N*_50_ length was 662 kbp, assuming a genome size of 6.3 Mbp. The G+C content of this bacterium (60.70%) was similar to that of other sequenced *Pseudomonas* genomes (9). Automated annotation using the NCBI Prokaryotic Genomes Automatic Annotation Pipeline (PGAAP) (http://www.ncbi.nlm.nih.gov/genomes/static/Pipeline.html) assigned a total of 5,533 genes, of which 3,460 have a known function, with 9 rRNAs and 53 tRNAs.

By using the Pfam search domain and tBLASTn applications, genes coding for enzymes involved in β-oxidation (*fad*), fatty acid *de novo* synthesis (*fab*), and mcl-PHA precursor availability (*phaG* and *phaJ*) were mapped in different loci along the entire genome. A type II PHA locus including genes for synthesis (*phaC1* and *phaC2*), depolymerization (*phaZ*), regulation (*phaD*), and phasins (*phaF* and *phaI*) was identified.

The genes involved in the metabolic steps of glycerol catabolism were also investigated in relation to the potential for industrial exploitation of the waste glycerol derived from biodiesel plants for PHA synthesis. While the glycerol kinase (GlpK), the cytoplasmic-membrane-associated glyeraldehyde-3-phosphate (G3P) dehydrogenase (GlpD), and the transcriptional regulator GlpR (10, 11) were present, no *glpF* gene encoding the glycerol facilitator protein and involved in glycerol transport (GlpF) has been detected in the genome of *P. mediterranea* strain CFBP 5447^T^.

As previously described for *P. corrugata* strain CFBP 5454 (12), large sequences coding for putative nonribosomal peptide synthetases were identified using AntiSMASH version 2.0 (13). Since it is estimated that approximately 3 kb of DNA is required to code for each amino acid activation module of the CLP peptide moiety, it can be presumed that the corpeptin-cormycin nonribosomal peptide synthetase (NRPS) system encompasses approximately 75 kb and 27 kb of DNA to encode their 22- and 9-amino-acid activation modules, respectively.

The release of the genome sequence of *P. mediterranea* strain CFBP 5447^T^ will enable researchers to improve the cost-effectiveness of bioprocesses.

### Nucleotide sequence accession numbers.

The draft genome sequence of *P. mediterranea* strain CFBP 5447^T^ has been deposited at DDBJ/EMBL/GenBank under the accession no. AUPB00000000. The version described in this paper is version AUPB01000000.
